# Roles of Akt and SGK1 in the Regulation of Renal Tubular Transport

**DOI:** 10.1155/2015/971697

**Published:** 2015-09-28

**Authors:** Nobuhiko Satoh, Motonobu Nakamura, Masashi Suzuki, Atsushi Suzuki, George Seki, Shoko Horita

**Affiliations:** ^1^Department of Nephrology, The University of Tokyo Hospital, 7-3-1 Hongo, Bunkyo, Tokyo 113-8655, Japan; ^2^Yaizu City Hospital, 1000 Dobara, Yaizu, Shizuoka 425-8505, Japan

## Abstract

A serine/threonine kinase Akt is a key mediator in various signaling pathways including regulation of renal tubular transport. In proximal tubules, Akt mediates insulin signaling via insulin receptor substrate 2 (IRS2) and stimulates sodium-bicarbonate cotransporter (NBCe1), resulting in increased sodium reabsorption. In insulin resistance, the IRS2 in kidney cortex is exceptionally preserved and may mediate the stimulatory effect of insulin on NBCe1 to cause hypertension in diabetes via sodium retention. Likewise, in distal convoluted tubules and cortical collecting ducts, insulin-induced Akt phosphorylation mediates several hormonal signals to enhance sodium-chloride cotransporter (NCC) and epithelial sodium channel (ENaC) activities, resulting in increased sodium reabsorption. Serum- and glucocorticoid-inducible kinase 1 (SGK1) mediates aldosterone signaling. Insulin can stimulate SGK1 to exert various effects on renal transporters. In renal cortical collecting ducts, SGK1 regulates the expression level of ENaC through inhibition of its degradation. In addition, SGK1 and Akt cooperatively regulate potassium secretion by renal outer medullary potassium channel (ROMK). Moreover, sodium-proton exchanger 3 (NHE3) in proximal tubules is possibly activated by SGK1. This review focuses on recent advances in understanding of the roles of Akt and SGK1 in the regulation of renal tubular transport.

## 1. Introduction

Renal tubules reabsorb most of water and electrolytes such as sodium, bicarbonate, and phosphate from glomerular filtrate. In particular, sodium reabsorption is regulated by various transporters along the nephron and to elucidate the mechanism of its regulation is important because excess renal sodium retention causes hypertension in metabolic syndrome that leads to cardiovascular disease [1]. Several hormonal signaling pathways have been shown to be involved in the expression and activation of such renal tubular transporters.

Akt is a serine/threonine kinase that was initially identified as an oncogene, as suggested by its name (formed from AK mouse + transforming or thymoma) [2]. It is also known as protein kinase B (PKB). Insulin stimulates phosphorylation of Akt and activated Akt exerts various biological effects such as cell growth and survival, angiogenesis, and metabolism by regulating downstream effectors [3]. For example, Akt induces the translocation of glucose transporter 4 (GLUT4) from intracellular sites to the plasma membrane, triggering glucose uptake into muscle and adipocytes. The insulin-induced Akt activation also impacts renal tubular transport through several signaling pathways and thus regulates sodium reabsorption.

SGK1 was initially discovered as a serum- and glucocorticoid-inducible serine/threonine kinase in rat mammary tumor cells [4, 5]. It is ubiquitously expressed in most tissues under the control of hormones such as glucocorticoids and mineral corticoids and regulated at the transcriptional level by osmotic changes [6]. Similar to Akt, SGK1 is also known to be a powerful regulator of metabolism, transcription, enzyme activity, and renal transport [7]. Furthermore, insulin also has stimulatory effects on SGK1 and activated SGK1 acts upon renal tubular transport independently or sometimes cooperatively with Akt [8]. In particular, SGK1 has a key role in NaCl homeostasis through regulating the epithelial sodium channel (ENaC) [9] in cortical collecting ducts (CCDs). Other than that, it stimulates a large number of renal tubular transporters, maintaining electrolyte balance.

In this review, we focus on the role of Akt- and SGK1-mediated signaling pathways in the regulation of renal tubular transport, especially sodium homeostasis, with emphasis on recent advances.

## 2. Akt Signaling

### 2.1. Akt: Isoforms, Distribution, and Structures

**Figure 1:**
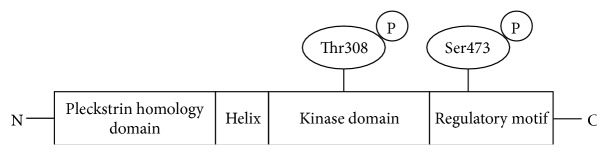
Schematic structure of Akt1. Akt1 has 480 amino acids and it is composed of four domains: the pleckstrin homology domain, the helix domain, the kinase domain, and the regulatory domain. The kinase and the regulatory domains have phosphorylation sites at Thr308 and Ser473, respectively. Phosphorylation of these sites induces Akt1 activation.

Akt has three isoforms, Akt1, Akt2, and Akt3. Akt1 is widely expressed in most tissues and has been implicated in cell growth and survival. In contrast, the expression patterns and functions of Akt2 and Akt3 are more limited. Akt2 is highly expressed in insulin-sensitive tissues such as skeletal muscle and adipocytes, where it mediates insulin-induced glucose uptake [10]. Akt3 is predominantly expressed in brains and testes, where it may be involved in pathophysiology of neurological disorders [11]. Akt1, Akt2, and Akt3 are encoded by distinct loci located on 14q32.3, 19q13.2, and 1q43-q44, respectively. All of the Akts have similar structures that include a pleckstrin homology (PH) domain, a helix region, a kinase domain, and a regulatory motif as shown in Figure 1 [11]. Human Akt1 has key phosphorylation sites, Thr308 and Ser473 in the kinase domain and the regulatory motif, respectively. Similarly, Akt2 and Akt3 also have phosphorylation sites at homologous positions.

### 2.2. Akt in Insulin Signaling Pathway

**Figure 2:**
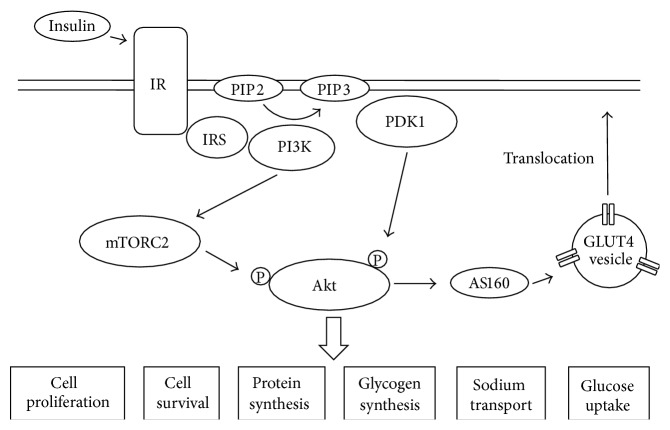
Insulin-mediated Akt signaling. Insulin binding to insulin receptor induces Akt activation through IRS/PI3K pathway. mTORC2 also activates Akt. Activated Akt regulates various biological functions such as cell survival and proliferation, protein synthesis, glycogen synthesis, sodium transport, and glucose uptake through translocation of GLUT4. IR: insulin receptor, IRS: insulin receptor substrate, PI3K: phosphatidylinositol 3-kinase, PDK1: phosphoinositide-dependent protein kinase 1, and mTORC2: mammalian target of rapamycin complex 2.

Among three Akt isoforms, Akt2 is essential for insulin-mediated glucose uptake into muscle and adipocytes. Binding of insulin to the insulin receptor (IR) first activates intrinsic receptor kinase function, resulting in phosphorylation of several key sites on the intracellular domain of the receptor. Insulin receptor substrate (IRS) binds to these phosphorylated sites on IR and in turn is activated via phosphorylation. Activated IRS protein binds to phosphatidylinositol 3-kinase (PI3K) that converts phosphatidylinositol (4,5)-bisphosphate (PIP2) to phosphatidylinositol (1,4,5)-triphosphate (PIP3). PIP3 facilitates translocation of phosphoinositide-dependent protein kinase 1 (PDK1) and Akt2 close to the membrane for activation of PDK1 and Akt2. In addition, activated PDK1 phosphorylates Akt2 at Thr309. On the other hand, PI3K also activates another mediator, the mammalian target of rapamycin complex 2 (mTORC2) which phosphorylates Akt2 at Ser474 [10]. Eventually, activated Akt2 induces the translocation of GLUT4 from intracellular sites to the plasma membrane, triggering glucose uptake into muscle and adipocytes. The insulin/Akt signaling cascade including Akt2-mediated GLUT4 translocation is summarized in Figure 2.

### 2.3. Akt and Insulin-Induced GLUT4 Translocation

GLUT4 is a member of the facilitative glucose transporter family that is predominantly expressed in muscle and fat tissues [12]. GLUT4 may also be expressed in renal tubules [13]; however, its detailed distribution in the kidney has not yet been clarified. Although there is a dispute regarding the role of Akt2 in the translocation of GLUT4 to the plasma membrane, Gonzalez and McGraw showed that Akt2 is indispensable for the insulin-stimulated initiation of GLUT4 translocation. By pharmacological inhibition or knockdown with short hairpin RNA, they showed that the Akt substrate AS160 is a key factor in GLUT4 translocation [14].

### 2.4. Akt and Tubular Transport

Proximal tubules (PTs) reabsorb most of water, nutrients, and electrolytes such as glucose, sodium, bicarbonate, and phosphate from glomerular filtrate. In the PTs, sodium is reabsorbed primarily by sodium-proton exchanger 3 (NHE3) on the apical side and by sodium-bicarbonate cotransporter (NBCe1) on the basolateral side. Several hormones and mediators such as angiotensin II, nitric oxide, dopamine, and insulin are known to regulate these transporters. [1, 15]. Among them, insulin has previously been shown to stimulate PT sodium and volume transport [16, 17].

By using IRS1^−/−^ and IRS2^−/−^ mice, we previously showed that the IRS2/PI3K pathway plays a major role in the stimulation of renal proximal absorption by insulin [18]. Additionally, by gene silencing in rats with short interfering RNA (siRNA) against IRS1 or IRS2, we also showed that while IRS2 mediates the stimulatory effect of insulin on PT transport, IRS1 mediates the stimulatory effect of insulin on glucose uptake into adipocytes [19].

Interestingly, we also found that while the stimulatory effect of insulin on glucose uptake and Akt phosphorylation in adipocytes were markedly reduced, the insulin-induced stimulation of NBCe1 and Akt phosphorylation in kidney cortex were completely preserved in insulin resistant human patients and Otsuka Long-Evans Tokushima Fatty (OLETF) rats [19]. In insulin resistance, the IRS2 in kidney cortex is exceptionally preserved and may mediate the stimulatory effect of insulin on NBCe1.

**Figure 3:**
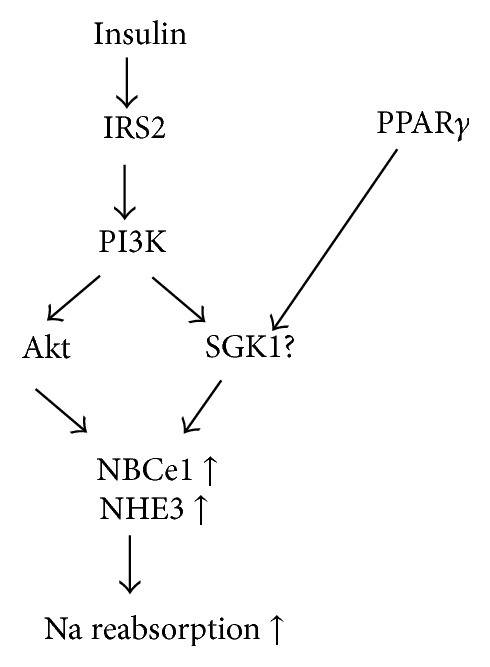
Effects of insulin on PT sodium transport. Insulin stimulates NBCe1 and NHE3 via IRS2/PI3K/Akt pathway. PPAR*γ* and SGK1 may be involved in the stimulatory effect on PT sodium transport. NBCe1: sodium-bicarbonate cotransporter 1, NHE3: sodium-proton exchanger 3, PPAR*γ*: peroxisome proliferator-activated receptor gamma, and SGK1: serum- and glucocorticoid-inducible kinase 1.

These findings strongly suggest that hyperinsulinemia in insulin resistance induces volume expansion and hypertension via PT transport stimulation and Akt mediates the stimulatory effect of insulin on NBCe1 as a downstream effector of insulin/IRS2/PI3K pathway. Akt2 is reported to be involved in the regulation of sodium-coupled glucose transport [20] and sodium-coupled phosphate transport [21]. SGK1 may be also involved in the regulation of PT transport as discussed below. Thus, further investigation would be required to elucidate how Akt actually mediates the insulin action on PT transport. The effect of insulin on PT is summarized in Figure 3.

### 2.5. Akt, With-No-Lysine Kinases (WNKs), and Sodium-Chloride Cotransporter (NCC)

Sodium-chloride cotransporter (NCC) is primarily expressed in distal convoluted tubules and mediates sodium and chloride reabsorption [22]. It is under the control of several hormones such as insulin, angiotensin II, glucocorticoids, and aldosterone [22]. Among these hormones, aldosterone has previously been recognized to stimulate NCC activity but the detailed mechanism of this process is only now being clarified. The discovery of with-no-lysine kinases (WNKs) [23] may provide a clue for deciphering this complicated mechanism.

WNK has several isoforms and variants including WNK1 (chromosome 12p13.3), WNK2 (chromosome 9q22.31), WNK3 (chromosome Xp11.22), WNK4 (chromosome 17q21.31), and kidney-specific WNK1 (KS-WNK1). In contrast to the other isoforms, the significance of WNK2 has not been established [24]. The physiological and pathological roles of WNKs are described in excellent reviews, for example, those by Hoorn and Ellison and by Uchida [25, 26].

In distal convoluted tubule (DCT) cells, WNKs play crucial roles in the regulation of NCC. Along with phosphorylating STE20/SPS1-related proline/alanine-rich kinase (SPAK) and oxidative-stress-responsive kinase 1 (OSR1), WNK3 induces NCC translocation from cytosol to luminal membrane. Phosphorylated OSR1/SPAK then activates NCC by phosphorylation (WNK-OSR1/SPAK-NCC phosphorylation cascade). In contrast, WNK4 had been shown to inhibit NCC by promoting lysosomal degradation [22, 25]. However, Takahashi and colleagues recently showed that insulin fails to stimulate NCC in WNK4^−/−^ mice [27]. Moreover, these authors found that transgenic mice overexpressing WNK4 exhibit pseudohypoaldosteronism type II (PHA-II) [28], suggesting that WNK4 positively regulates NCC. Further experiments would be required to establish the precise role of WNK4 in the regulation of NCC.

Recently, Akt has been suggested to mediate insulin-induced WNK-OSR1/SPAK-NCC phosphorylation cascade. In* db/db* diabetic mice that show hyperinsulinemia and high thiazide sensitivity, the phosphorylation of renal Akt as well as NCC is increased. Furthermore, this increased phosphorylation of NCC is corrected by specific inhibitors for PI3K or Akt, suggesting that the insulin/PI3K/Akt pathway regulates WNK-OSR1/SPAK-NCC phosphorylation cascade [29]. Insulin also induces strong renal Akt phosphorylation in another model of metabolic syndrome Zucker obese rats, resulting in NCC phosphorylation [30]. The stimulation of NCC may be also involved in hyperinsulinemia-induced hypertension observed in metabolic syndrome.

## 3. SGK Signaling

### 3.1. SGK: Isoforms, Distribution, and Structures

To date, SGK1 is known to have two isoforms, SGK2 and SGK3. Like SGK1, SGK3 is ubiquitously expressed in a variety of tissues; however, the expression of SGK2 is limited to liver, kidney, pancreas, and brain. In human, SGK1, SGK2, and SGK3 are encoded by different loci, 6q23, 20q12, and 8q12.3, respectively. About 80% of amino acids in catalytic domain of SGK2 and SGK3 are identical to that of SGK1 [31].

### 3.2. SGK1 in Insulin Signaling Pathway

**Figure 4:**
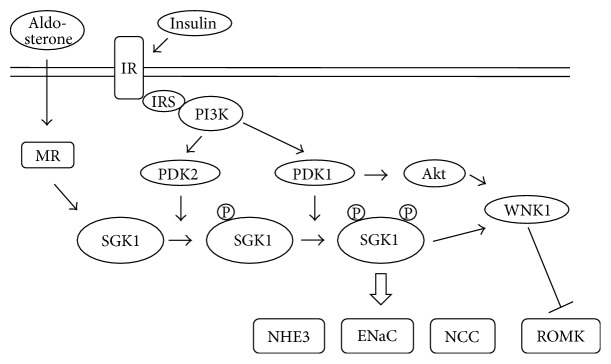
SGK1-mediated signaling pathway. Aldosterone stimulates SGK1 via mineralocorticoid receptor (MR), while insulin stimulates SGK1 via IRS/PI3K pathway. SGK1 is phosphorylated twofold by PDK1 and PDK2. Activated SGK1 exerts stimulatory effects on sodium transport through ENaC, NCC, and NHE3. Also, SGK1 regulates potassium secretion through ROMK. SGK1: serum- and glucocorticoid-inducible kinase 1, IRS: insulin receptor substrate, PI3K: phosphatidylinositol 3-kinase, PDK1: phosphoinositide-dependent protein kinase 1, WNK1: with-no-lysine kinase 1, ENaC: epithelial Na^+^ channel, ROMK: renal outer medullary potassium channel, and NHE3: sodium-proton exchanger 3.

Insulin can stimulate SGK1 through insulin/IRS/PI3K pathway. In the insulin/PI3K pathway, SGK1 is phosphorylated twofold by PDK1 and PDK2. Thus, activated SGK1 exerts a variety of effects on renal transporters, including the stimulation of ENaC, NCC, and NHE3 and the inhibition of renal outer medullary potassium channel (ROMK) through phosphorylation of WNK1, as will be discussed below [8, 31, 32].  Figure 4 summarizes the typical signal transduction of SGK1.

### 3.3. SGK1 and Tubular Transport

SGK1 is well known to be an efficient regulator of ENaC [9] in CCD. It activates ENaC through several pathways [33] and among these pathways, inhibition of E3 ubiquitin ligase, neural precursor cell-expressed, developmentally downregulated protein 4-2 (Nedd4-2) seems to be the most important.

Nedd4-2 binds to and ubiquitinates ENaC at the cell surface, triggering its internalization and degradation [34]. SGK1 phosphorylates and inhibits Nedd4-2, resulting in increased expression level of ENaC. In fact, kidney tubule-specific SGK1 knockout mice [35] exhibit decreased phosphorylation of Nedd4-2 and decreased expression of all ENaC subunits. Moreover, expression of NCC in the kidney was decreased. These findings suggest that NCC is also regulated by SGK1 [36, 37]. Ronzaud and colleagues showed that nephron-specific Nedd4-2-deficient mice exhibit salt-dependent hypertension due to the upregulation of NCC [38]. Therefore, Nedd4-2 may be involved in inhibition of both ENaC and NCC. On the other hand, WNK4, which is phosphorylated by SGK1 at serine 1169, is also involved in the regulation of ENaC. [39].

Recently, SGK1 is also suggested to modulate PT transport. For example, SGK1 has been shown to be upregulated by angiotensin II, which results in the activation of NHE3 and increased PT sodium reabsorption [32, 40, 41]. Fuster and colleagues also showed that SGK1 mediates the chronic stimulatory effect of insulin on NHE3 in a cell line model of PT [17]. Moreover, SGK1 might mediate NHE3 activation via peroxisome proliferator-activated receptor gamma (PPAR*γ*) in human PT cells [40]. Another transporter in PT, sodium-dicarboxylate cotransporter, is also upregulated by both SGK1 and Akt [42].

## 4. Cross-Talk between Akt and SGK1 in the Regulation of ROMK

ROMK, also called Kir1.1 [43, 44], regulates potassium secretion in the outer medullary collecting duct, thereby playing an important role in the homeostasis of potassium. Interestingly, both Akt1 and SGK1 regulate the expression of ROMK. Cheng and Huang [8] found that PI3K-activating ligands like insulin or IGF-1 inhibited ROMK by facilitating its endocytosis via phosphorylation of Akt, SGK1, and WNK1. Knockdown of WNK1 prevented the inhibitory effect of insulin on ROMK, and phosphorylation at Thr58 of WNK1 was indispensable for the insulin effect. The authors concluded that both Akt and SGK1 can cooperatively inhibit ROMK through WNK1 phosphorylation.

## 5. Recent Advances in the Clinical Development of Akt and SGK Inhibitors

Akt, as mentioned above, plays an important role in signaling pathways of cell growth, cell survival, glucose metabolism, and renal tubular transport. In particular, several lines indicate that Akt isoforms are overexpressed or hyperactivated in a broad range of human cancers through dysregulated activation of PI3K/Akt pathway. Furthermore, the activation of Akt is shown to be related with resistance to chemotherapy or radiotherapy and thus involved in the progression or poor prognosis in some types of tumors [45, 46]. Therefore, Akt and other components in upstream or downstream of PI3K pathway are expected to be a key target for therapeutic intervention [47].

Several drugs targeting each component in PI3K/Akt pathway are now being investigated in a variety of tumors [48, 49]. For example, an allosteric Akt inhibitor, MK-2206, has been reported to exert potent inhibitory effect on tumor growth in combination with other cytotoxic agents or receptor tyrosine kinase inhibitors and recently entered human clinical trials. Phase II study of MK-2206 as a single agent has revealed that it has favorable toxicity profile with a mild response in lymphoma and further study for mechanism-based combination therapy would be required [50].

SGK1 also regulates cell growth and survival as a downstream effector of PI3K pathway, and it is overexpressed in some types of tumors including colon cancer, medulloblastoma, prostate cancer, ovarian tumors, and non-small cell lung cancer, but not in all tumors [51, 52]. A previous study showed that* Sgk1* (−/−) mice exhibited significantly fewer colonic tumors than* Sgk1* (+/+) mice after chemical cancerogenesis, suggesting the therapeutic possibility of SGK1 inhibitors [53]. Indeed, a highly selective SGK1 inhibitor, EMD638683, has been shown to suppress colonic tumor growth in vivo [54]. Furthermore, GSK650394 that functionally inhibits enzymatic activity of SGK1 attenuated androgen-mediated growth of the prostate cancer cell line [55].

Thus, components in PI3K pathway contribute to the development of a variety of cancers and selective use of different inhibitors in the pathway would be expected in cancer therapeutics. On the other hand, the modulators of Akt and SGK1 as the therapeutics for diabetes mellitus and other metabolic diseases have not been developed.

## 6. Conclusion

**Figure 5:**
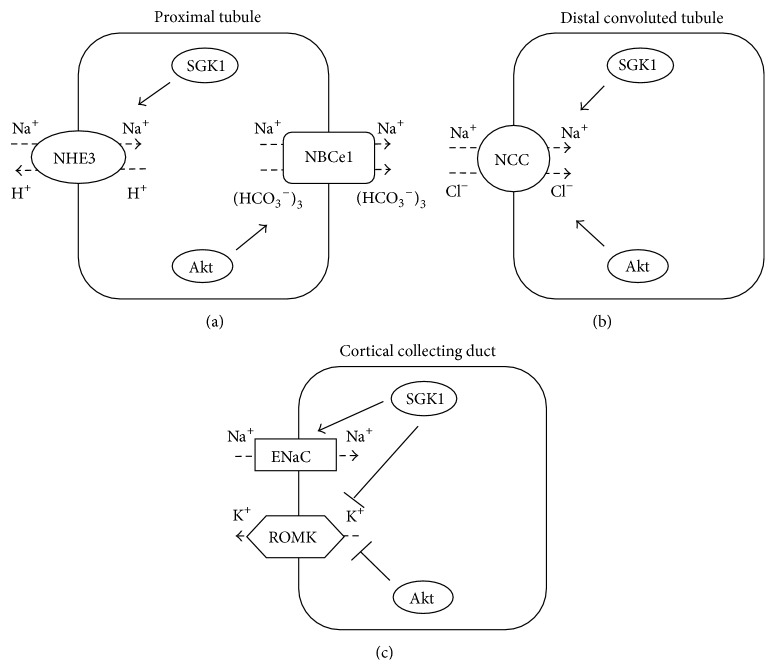
Roles of Akt and SGK1 in transport regulation at each nephron segment: Akt and SGK1 modify renal tubular transport by activating or inhibiting several transporters at each nephron segment. Targets and functions of the kinases in proximal tubules, distal convoluted tubules, and cortical collecting ducts are shown in (a), (b), and (c), respectively.

In this review, we have summarized the roles of Akt and SGK1 in renal tubular transport. As a downstream effector of insulin/IRS/PI3K cascade, Akt stimulates sodium reabsorption in several nephron segments. On the other hand, SGK1, activated by PI3K-mediated signaling pathway, also acts upon renal tubular transport independently or sometimes cooperatively with Akt.  Figure 5 summarizes the roles of Akt and SGK1 in transport regulation at each nephron segment.

In PTs, Akt stimulates NBCe1 to increase sodium reabsorption and SGK1 possibly activates NHE3. In DCTs, PI3K/Akt signaling pathway is thought to activate WNK-OSR1/SPAK-NCC phosphorylation cascade to stimulate NCC. It is presumed that SGK1 also mediates NCC activation in part; however, the precise mechanism has not been clarified. In CCDs, SGK1 regulates the expression level of ENaC through inhibition of its degradation by Nedd4-2. On the other hand, both Akt and SGK1 are involved in insulin-mediated ROMK inhibition via phosphorylation of WNK1.

However, how Akt and SGK1 actually regulate the activation of those transporters has not yet been clarified. Furthermore, the precise interaction between Akt and SGK1 in renal tubules also remains to be determined.
